# Visceral regeneration in a sea cucumber involves extensive expression of *survivin *and *mortalin *homologs in the mesothelium

**DOI:** 10.1186/1471-213X-10-117

**Published:** 2010-11-29

**Authors:** Vladimir S Mashanov, Olga R Zueva, Carmencita Rojas-Catagena, Jose E Garcia-Arraras

**Affiliations:** 1 Department of Biology, University of Puerto Rico, PO Box 70377, San Juan, PR 00936-8377, USA

## Abstract

**Background:**

The proper balance of cell division and cell death is of crucial importance for all kinds of developmental processes and for maintaining tissue homeostasis in mature tissues. Dysregulation of this balance often results in severe pathologies, such as cancer. There is a growing interest in understanding the factors that govern the interplay between cell death and proliferation under various conditions. *Survivin *and *mortalin *are genes that are known to be implicated in both mitosis and apoptosis and are often expressed in tumors.

**Results:**

The present study takes advantage of the ability of the sea cucumber *Holothuria glaberrima *Selenka, 1867 (Holothuroidea, Aspidochirota) to discard its viscera and completely regrow them. This visceral regeneration involves an extensive expression of *survivin *and *mortalin *transcripts in the gut mesothelium (the outer tissue layer of the digestive tube), which coincides in time with drastic de-differentiation and a burst in cell division and apoptosis. Double labeling experiments (in situ hybridization combined with TUNEL assay or with BrdU immunohistochemistry) suggest that both genes support cell proliferation, while *survivin *might also be involved in suppression of the programmed cell death.

**Conclusions:**

Visceral regeneration in the sea cucumber *H. glaberrima *is accompanied by elevated levels of cell division and cell death, and, moreover, involves expression of pro-cancer genes, such as *survivin *and *mortalin*, which are known to support proliferation and inhibit apoptosis. Nevertheless, once regeneration is completed and the expression pattern of both genes returns to normal, the regrown digestive tube shows no anomalies. This strongly suggests that sea cucumbers must possess some robust cancer-suppression mechanisms that allow rapid re-growth of the adult tissues without leading to runaway tumor development.

## Background

The ability of echinoderms to repair their injured or autotomized body parts has been well known [1,2]. One of the examples of such a remarkable capacity is rapid and complete regeneration of the digestive tube in holothurians (sea cucumbers) following induced or spontaneous evisceration (= autotomy of the viscera). Evisceration results in the loss of the entire digestive tube, except for small regions of the esophagus and cloaca (in some species, however, the esophagus and the pharynx are lost as well) [3,4]. It has been shown that visceral regeneration in sea cucumbers is accomplished by massive remodeling of the remaining tissues of the mesentery and of the cloacal and esophageal stumps through a complex combination of morphogenetic events including de-differentiation of specialized cells, their migration, cell death, cell division, and re-differentiation [5-9]. Therefore, the regenerating digestive tube of sea cucumbers provides a unique experimental model for studying processes of extensive cell activation and proliferation without uncontrolled tumor formation. Another experimental advantage of this system is that the injury occurs by autotomy in pre-determined regions [3,4], i.e., in a very consistent and repeatable manner, which excludes variation between animals in the extent and severity of the trauma. However, the molecular machinery underlying such an extraordinary plasticity in post-embryonic tissues remains largely unknown.

Post-traumatic regeneration, as any other developmental process, requires a tightly regulated interplay between cell proliferation and cell death. The present paper deals with two of the genes, *survivin and mortalin*, that are known to play a dual role in regulation of both programmed cell death and cell division in diverse groups of animals [10-14]. Although there are plenty of data on the involvement of *survivin *and *mortalin *in malignant diseases [12,13,15-17], only rare studies have directly dealt with the functional significance of these two genes in post-traumatic regeneration [18-20].

Survivin (also known as BIRC5) is a small multifunctional protein, a member of the inhibitor of apoptosis (IAP) protein family [12]. IAPs are defined by the presence of the well-conserved baculovirus IAP repeat (BIR) domain [21,22], which functions as a protein interaction module consisting of about 70 amino acids. This feature allows IAPs to control a wide variety of cellular pathways through cooperation with other polypeptides. Survivin interacts with many proteins that are important for regulation of both cell death and cell division. For instance, like many other BIR-containing proteins, survivin suppresses apoptosis. After forming a complex with the co-factor protein HBXIP (hepatitis B X-interacting protein), survivin specifically binds pro-caspase 9, an initiator protease of the mitochondria-dependent apoptosis pathway [23]. Survivin also plays an important role in cell division and its expression was reported to overlap with several stem cell markers [24]. Through interaction with Ran, survivin protein regulates mitotic spindle formation [25]. In association with the proteins INCENP, aurora B, and borealin, survivin forms the multiprotein chromosomal passenger complex, which plays multiple roles in cell division, being involved, for instance, in correction of kinetochore attachment errors, assembly/stabilization of microtubules of the mitotic spindle, and completion of cytokinesis [26].

The *survivin *expression levels is usually high in most human cancers studied so far, but is largely absent from normal adult tissues, with a few notable exceptions, including the gastric mucosa, thymus, placenta, and testes [13,27,28]. Increased expression of *survivin *in cancer patients is considered an unfavorable prognostic marker correlating with decreased survival chances, risk of recurrence, metastasis, and resistance to anti-cancer drugs [13,29]. During embryogenesis, *survivin *is prominently expressed in various (although not all) embryonic tissues, including the gastrointestinal tract, neural tube and blood vessels, in a developmentally regulated stage-dependent fashion, and knockdown experiments suggest that it is involved in regulation of neurogenesis, angiogenesis and hematopoiesis [30-32].

Mortalin (also known as Hspa9, Grp75, and PBP74) is a heat un-inducible member of Hsp70 family of proteins, which is cable of interacting with a variety of binding partners and performing various functions [14]. Like all Hsp70 family chaperones, mortalin is composed of two domains: an N-terminal nucleotide-binding (ATPase) domain and a C-terminal substrate binding domain [14]. As a chaperone, mortalin binds misfolded proteins and assists them to reach their functional configuration through ATP-dependent conformational change [33]. It is also involved in stress response and intracellular trafficking [14]. But most interestingly, mortalin is known to perform functions related to the control of cell proliferation and survival. It is known to bind the tumor suppressor protein p53 and therefore prevents the latter from inducing apoptosis and inhibiting cell division [34-36]. Elevated mortalin expression has been observed in many human tumors, with higher levels of mortalin expression corresponding to more aggressive tumor phenotypes [14,16,17]. Conversely, downregulation of mortalin expression was demonstrated to suppress the growth of human transformed cells [16].

Previous studies have demonstrated that successful visceral regeneration in sea cucumbers is accomplished through extensive proliferation of the tissues of the mesentery and the stumps of the gut [5,7-9]. Cell death, however, has never been studied, although it is known to be equally important in regeneration [37,38]. Here, we report that regeneration of the digestive tube in the sea cucumber *Holothuria glaberrima *involves a significant increase in cell death in comparison to non-injured animals and provide a detailed description of spatio-temporal expression pattern of *survivin *and *mortalin *transcripts.

## Methods

### Animal collection, maintenance and evisceration

Adult individuals of the sea cucumber *Holothuria glaberrima *Selenka, 1867 (Holothuroidea, Aspidochirota) were collected at low tide from the rocky coast adjacent to Old San Juan, Puerto Rico and immediately transported to the laboratory were they were allowed to adapt to laboratory conditions for 16 - 24 h in aerated seawater (brought from the sampling site) at room temperature. Evisceration was induced by injecting a few milliliters of 0.35 M KCl into the coelomic cavity. Eviscerated animals were kept in well-aerated in-door seawater tanks. The well-being of animals was ensured by keeping the density of holothurians in the tanks at approximately one individual per liter seawater. Seawater was changed on days 3, 7, 14, and 21 after evisceration.

### Sequence analysis

The homologs of *survivin *and *mortalin *were identified among 5173 EST sequences representing three cDNA libraries from the normal and regenerating digestive tube of *H. glaberrima *[39] by BLAST query against the non-redundant protein database of the NCBI. To obtain full-length cDNA sequences, we performed 5' and 3' RACE using SMARTer RACE cDNA Amplification kit (Clontech) following the manufacturer's protocol. The resulting sequences of *survivin *and *mortalin *of *H. glaberrima *were deposited into the GenBank under accession numbers HQ174778 and HQ174779, respectively. Conserved domain search was performed by analyzing the predicted survivin and mortalin protein sequences with the online protein domain prediction program SMART [40] and InterProScan [41]. Coiled-coil regions were predicted using the COILS server at EMBnet (http://www.ch.embnet.org/software/COILS_form.html). Further sequence analysis was performed by aligning the predicted protein sequences of survivin and mortalin with the corresponding orthologs from other deuterostomes using ClustalX, version 2.0.10 [42]. Jalview version 2.4.0.b2 [43] was used for analysis of multiple sequence alignments.

### Real-time quantitative RT-PCR

The non-eviscerated and regenerating individuals of *H. glaberrima *were anesthetized in 0.2% cholobutanol (1,1,1-trichloro-2-methyl-2-propanol hydrate) (Sigma) in seawater for 15 - 30 min at room temperature. In order to prevent possible RNA degradation during the subsequent dissection, the sedated holothurians were placed in ice-cold sea water and all manipulations were performed as quickly as possible. The animals were cut open along the dorsal interambulacrum, the normal gut or the anterior and posterior regenerates were excised, and immediately immersed in RNAlater (Ambion). Total RNA was isolated using TRI regent (Sigma) and treated with RNAse-free DNAse I (Qiagen) to minimize the noise due to possible genomic DNA contamination. First-strand cDNA was synthesized from 1 μg of the total RNA with random hexamer primers and ImPromt-II reverse transcriptase (Promega). PCR primers were designed using Primer Premier 5.0 software (PREMIER Biosoft International), and their sequences are shown in Additional File 1. qPCR reactions were set up in a reaction volume of 20 μl using PerfeCta SYBR Green Fast Mix (Quanta Biosciences) with the final concentration of the PCR primers of 200 nM and were then run on an iCycler iQ PCR Detection System (Bio-Rad) with the following parameters: 95°C for 10 min (denaturation step) followed by 45 cycles of 95°C for 30 sec, 55°C for 30 sec, and 72°C for 30 sec (amplification step). Fluorescence data were collected during the 72°C incubation phase. After amplification, melting curve analysis (55 - 95°C with a heating rate of 0.1°C/sec and a continuous fluorescence measurement) was performed for each of the PCR products to ensure the specificity of the reaction. Real-time PCR reactions were performed on three independent RNA samples purified from each of the regeneration stages as well as from the normal gut (biological replicates). All samples were analyzed in triplicate (technical replicates). The relative expression values of *survivin *and *mortalin *were normalized relative to the expression of the housekeeping gene *NADH dehydrogenase subunit 5 *using equations from [44]. To determine the real-time PCR efficiencies, serial two-fold dilutions of cDNA templates were run in triplicates in a PCR reaction. The corresponding real-time PCR efficiencies (E) were calculated from the slope values produced by the iCycler software according to the equation: E = 10^(-1/slope) ^[44]. The investigated transcripts showed amplification efficiencies of 2.00, 2.018, and 1.99, for *mortalin*, *survivin*, and *NADH dehydrogenase subunit 5*, respectively, with high linearity (correlation coefficient R ≥ 0.992).

### In situ hybridization

DIG-labeled riboprobes for in situ hybridization were synthesized from PCR-generated DNA templates. Briefly, RNA isolation and cDNA synthesis were performed as described above. The cDNA was amplified in a PCR reaction with gene-specific primers (without the RNA polymerase promoters at this stage) (Additional File 1) to generate what we call a pre-template for each of the genes of interest. The specificity of this PCR product was confirmed by direct sequencing. In the second set of PCR reactions, the templates were generated by amplifying the pre-templates with the appropriate primer (the reverse primer for the antisense probes, and the forward primer for the sense probes) containing the T7 RNA promoter sequence at the 5' end. The PCR products were then gel purified and used as templates to transcribe riboprobes with DIG RNA Labeling Kit (Roche) following the manufacturer's protocol. The *survivin *riboprobe targeted the last 20 nucleotides of the 5' UTR plus nucleotides 1 - 184 of the ORF. The *mortalin *riboprobe spanned nucleotides 1919 through 2353 of the ORF. Both antisense and sense probes were generated; the sense probes were used in the negative control reactions, and none of them showed detectable hybridization signal.

In situ hybridization staining was largely performed according to Holland et al. [45] with some modifications. Briefly, the animals were dissected as described above. Immediately upon excision, the tissue samples were briefly rinsed in ice-cold RNAse-free 0.01 M PBS (pH 7.4, 1030mOsm) and fixed overnight at 4°C in a freshly prepare mixture of 4% paraformaldehyde and 0.1% glutaraldehyde in PBS. After fixation, the samples were kept in 70% ethanol at -20°C. When needed, the samples were transferred to RNase-free 96-well cell culture plates, rinsed in PBS containing 0.1% Tween-20 (PBST), treated with 7.5 μg/ml proteinase K (Roche) for 10 min, acetylated sequentially in 0.25% and 0.5% acetic anhydride in 0.1 M triethanolamine, 5 min each. Prehybridization was performed at 60°C for 2 h or longer in hybridization buffer containing 5× SSC, 0.1% Tween-20, and 0.4 mg/ml salmon sperm DNA (Invitrogen). The riboprobes were diluted in warm (60°C) hybridization buffer to a final concentration of about 400 ng/ml and denatured at 80°C for 5 min. The hybridization was carried out at 58°C overnight in a hybridization oven equipped with a rocking platform. Stringency washes included four changes of 50% formamide in 5× SSC at 60°C, 50 min in 5× SSC at 37°C, 50 min in 2× SSC, and 15 min in 0.1× SSC at 50°C. The samples were then equilibrated in Washing Buffer (Roche) for 15 min at room temperature, followed by a 30 min incubation in Blocking Solution (Roche). Alkaline-phosphatase-conjugated anti-DIG antibodies (Roche) were diluted 1:2000 in Blocking Solution and applied overnight at 4°C. Excess antibody was removed by four washes in Washing Buffer, 20 min each. The samples were then equilibrated in four 10 min changes of a detection buffer containing 100 mM Tris-HCl pH 9.5, 100 mM NaCl and 0.1% Tween-20. Color reaction was performed at room temperature in the dark in a staining solution containing 4.5 μl of NBT stock solution (Roche) and 3.5 μl BCIP stock solution (Roche) per 1 ml of the detection buffer. The staining was stopped by two 10 min washes in a solution containing 10 mM Tris-HCl pH 8.1 and 1 mM EDTA. The samples were then equilibrated in PBST, postfixed in a mixture of 4% paraformaldehyde and 0.1% glutaraldehyde in PBS, cryoprotected in 10%, 20%, and 30% buffered sucrose, and incubated overnight in a 1:1 mixture of the 30% sucrose and the cryoembedding OCT medium (Takara) at 4°C. The samples were then frozen in the pure OCT medium. Serial cryosections were cut with a Leica CM1850 cryostat, collected onto gelatine-coated slides, dried overnight at 42°C and mounted in a mixture of 7.5% gelatine and 50% glycerol in 0.1 M PBS (pH 7.4). The preparations were analyzed and photographed with a Nikion Eclipse 600 microscope equipped with DIC optics and a SPOT RT3 digital camera (Diagnostic Instruments, Inc.).

All micrographs in the present paper represent transverse sections, which were cut orthogonal to the main axis of the organs. All figures are orientated with the ventral side of the animal to the bottom.

### Quantification of apoptosis

Tissue samples were obtained as described above and fixed overnight in 4% paraformaldehyde in 0.01 M PBS. Apoptosis was quantified by terminal deoxynucleotidyl transferase-mediated dUTP nick end labeling (TUNEL) with a Fluorescein FragEl DNA Fragmentation Detection kit (Calbiochem, Cat. QIA 39) on cryosections of the normal and regenerating gut. The percentage of TUNEL-positive cells was calculated by 'manually' counting FITC-positive cells and DAPI-positive nuclei on micrographs obtained with a 40× objective and imported into Fiji image processing software (http://pacific.mpi-cbg.de) with the Cell Counter plugin installed. Cell counting was performed on at least five 10 μm-thick cryosections per animal, and at least three animals were used per regeneration stage.

### Double labeling: in situ hybridization combined with TUNEL assay or BrdU immunoistochemistry

In double labeling experiments, in situ hybridization was carried out first followed by either TUNEL assay of BrdU immunohistochemistry. The tissue processing was performed as described above, except that for the cell proliferation assay, the animals were injected with ~0.1 mg BrdU per animal 24 h before being sacrificed. To visualize BrdU incorporation, the sections of the in situ hybridization whole mounts were treated with 2 N HCl for 30 min at 37°C, the acid was neutralized with 0.1 M borate buffer (pH 8.5), followed by PBS washes, incubation in 0.1 M glycine (1 h) and 2% goat serum (1 h), and then the rat anti-BrdU antibody (GenWay, 20-783-71418) diluted at 1:400 were applied overnight at 4°C. Incubation in the goat anti-rat FITC conjugated secondary antibody (GenWay, 25-787-278232) diluted at 1:50 was performed for 1 h at RT.

### Statistical analysis

For evaluation of statistical differences between the non-eviscerated gut and the different stages visceral regeneration, we employed Welch's ANOVA and Welch's t-test, which do not assume samples having equal variances, and are, therefore, more suitable for biological samples of relatively small size than the ordinary Student's t-test [46]. All statistical analyses were performed in R package version 2.11.1 (http://cran.r-project.org/). All values are reported as mean ± standard error.

## Results

### Orthologs of *survivin *and *mortalin *in *H. glaberrima*

**Figure 1:**
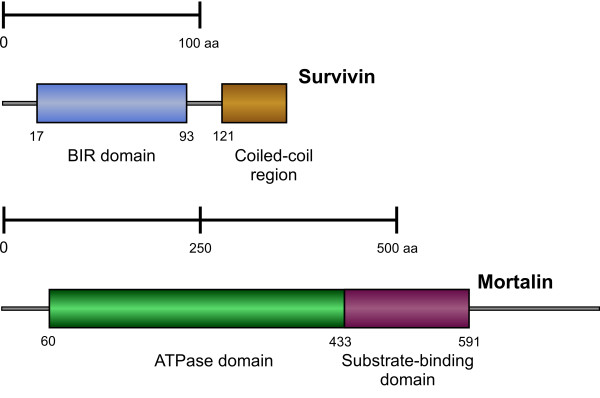
**Domain organization of the predicted survivin and mortalin proteins of *H. glaberrima***.

Sequences with significant (E value < 10^-30^) similarity to database entries for deuterostome orthologs of *survivin *and *mortalin *were identified in the cDNA library derived from the regenerating gut of *H. glaberrima*. The deduced protein sequence of *H. glaberrima survivin *(143 amino acids long) was found to have a single highly conserved BIR domain (17-93 aa) (Figure 1, Additional File 2). Moreover, computer analysis strongly suggests that the survivin protein of *H. glaberrima *has a left-handed coiled-coil region at its C-terminus (121-143 aa) (Figure 1). The BIR domain is known to be essential both for apoptosis inhibition and mitosis-related functions, while the coiled-coil motif is thought to allow survivin protein to interact with microtubules of the mitotic spindle [13,22]. The sea cucumber survivin protein exhibits 43-53% overall similarity with orthologs from other deuterostome species, with the greatest similarity (up to 69%) residing within the BIR domain (Additional files 2 and 3).

The predicted sequence of mortalin is 752 amino acids long. As all Hsp70 family members [14], the deduced sequence of *H. glaberrima *contains an N-teminal ATPase domain followed by a substrate-binding domain (Figure 1). This coincides with the fact that the chaperoning functions of mortalin require multiple binding and release of the substrate peptide and are ATP-dependent [14,33]. The sea cucumber mortalin protein shows a very high degree of identity (63.5 to 69.4%) with orthologs from other deuterostomes (Additional Files 4, 5, 6).

### Overview of the sea cucumber gut organization and evisceration phenomenon

**Figure 2:**
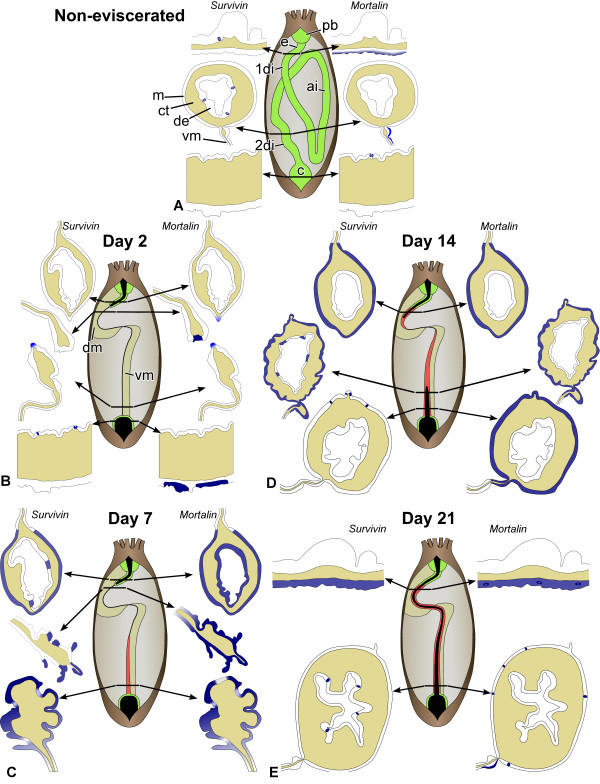
**Diagram summarizing the anatomical features of the non-eviscerated (normal) and regenerating digestive tube and the expression patterns of *survivin *and *mortalin *in *H. glaberrima***. **(A) **Non-eviscerated animals (on the anatomical drawing, the gut mesenteria are not shown). **(B) **- **(E) **Regenerating animals at day 2, 7, 14, and 21, respectively. 1di - first descending intestine; 2di - second descending intestine; ai - ascending intestine; c - cloaca; ct - connective tissue layer; de - digestive (luminal) epithelium; dm - dorsal mesentery; e - esophagus; m - mesothelium; pb - pharyngeal bulb; vm - ventral mesentery. All anatomical drawings are positioned with the anterior to the top. The arrows indicate the position of the representative transverse sections. Colors indicate the following: blue - in situ hybridization signal; green - non-eviscerated ('old') tissues; red - regenerating ('new') tissues; black - lumen of the digestive tube. Not to scale.

In order to make the reader familiar with the organization of the holothurian digestive system and the phenomenon of visceral regeneration, it may be helpful to provide a brief description here. For a more detailed reading, please refer to the previously published reviews and original papers (e.g., [1,5,8,47-49]). As in other sea cucumber species [47,49], the digestive tube of *H. glaberrima *consists of a pharynx, which lies within the so-called pharyngeal bulb, a short esophagus, an intestine, which is subdivided into the first descending, ascending and the second descending regions, and a cloaca (Figure 2A). The anterior regions of the digestive tube, including the esophagus and the first descending intestine, are suspended within the body cavity by the dorsal mesentery; the latter then continues into the lateral mesentery, which supports the ascending intestine, followed by the ventral mesentery attached to the second descending intestine in the posterior region of the body. The wall of the digestive tube consists of three histological layers: an inner digestive (luminal) epithelium, a connective tissue layer, and an outer mesothelium (also known as coelomic epithelium of the gut), which includes the gut musculature and a basiepithelial nervous plexus (Figure 2A). The gut mesothelium is continuous with the coelomic epithelia, which make up the mesentery.

Evisceration in *H. glaberrima *involves complete detachment of the intestine from the esophagus and the cloaca, and also from the mesentery [5]. The detached intestine, is then expelled through the rupture of the cloacal wall, and the wounds in the stumps of the esophagus and cloaca are initially sealed by muscular contraction before being healed.

### Spatiotemporal pattern of *survivin *expression

**Figure 3:**
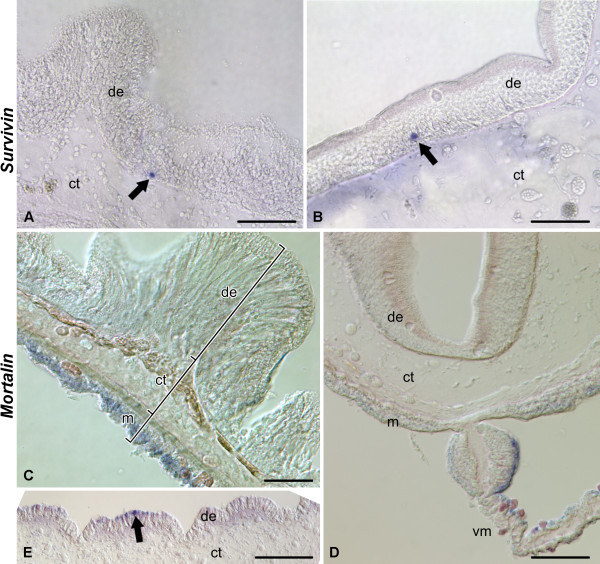
**In situ hybridization**. Expression of *survivin *(A and B) and *mortalin *(C - E) in the tissues of the digestive tube in non-eviscerated animals. **(A) **and **(B) ***survivin *expression in the scattered cells of the luminal epithelium in the esophagus **(A) **and the second descending intestine **(B)**. **(C) ***mortalin *transcripts widely expressed in the apical region of the mesothelium in the esophagus. **(D) **Asymmetric distribution of *mortalin *transcripts in the distal region of the mesentery attached to the second descending intestine. **(E) ***mortalin-*expressing cell in the luminal epithelium of the cloaca. Arrows indicate rare cells showing in situ hybridization signal. de - digestive (luminal) epithelium; ct - connective tissue layer; m - mesothelium; vm - ventral mesentery. Scale bars = 50 μm in **(A) **- **(C)**; 100 μm in **(D)**; 200 μm in **(E)**.

In the normal gut, *survivin *transcripts are detected by in situ hybridization in scattered, but strongly labeled cells, which are often spherical in shape and are mostly localized in the basal region of the luminal epithelium. No labeling is seen in the mesothelium nor in the connective tissue layer of the gut wall (Figure 2A; 3A, B).

**Figure 4:**
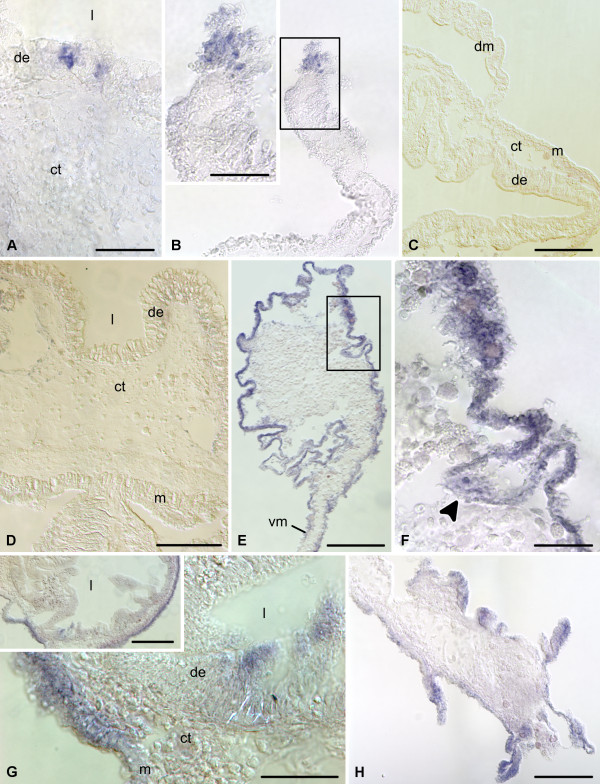
**In situ hybridization**. Expression of *survivin *at early stages of gut regeneration (days 2 - 6 after evisceration). **(A) **Digestive (luminal) epithelium of the cloaca on day 2. **(B) **Free distal edge of the posterior mesentery on day 2. The inset shows a higher magnification view of the boxed area. **(C) **The stump of the esophagus on day 2. **(D) **Wall of the cloaca on day 6. **(E) **A low-magnification view of the posterior regenerate on day 6. **(F) **Higher magnification of the boxed area on **(E) **showing a furrow (arrowhead) of the coelomic epithelium. **(G) **Esophageal stump on day 6. The inset shows a lower magnification view of the cross-section of the stump. **(H) **Free distal margin of the anterior mesentery on day 6. de - digestive (luminal) epithelium; dm - dorsal mesentery; ct - connective tissue layer; l - lumen of the gut; m - mesothelium; vm - ventral mesentery. Scale bars = 50 μm in **(A)**, **(B inset)**, **(F) **and **(G)**; 100 μm in **(B)**, **(G inset)**, and **(H)**; 200 μm in **(C) **- **(E)**.

On days 2-3 following evisceration, the wound at the anterior end of the cloacal stump is healed. In the luminal epithelium of this region, strongly labeled scattered cells can be observed quite regularly (Figure 2B, 4A). Strong expression of *survivin *is also observed in the coelomic epithelium of the mesentery that runs forward from the cloaca. Interestingly, this expression is limited to a group of cells at the free margin of the mesentery and is absent from other areas (Figure 2B, 4B). The remnant (stump) of the esophagus at the anterior end of the animal also seals its wound at the autotomy plane, but no in situ hybridization signal is seen either in the stump, or in the anterior mesentery (Figure 2B, 4C).

On days 6-7 after evisceration, no *survivin *transcripts are detected in the cloaca (Figure 4D). At this stage, a developing posterior rudiment can be clearly distinguished as a solid rod-like connective-tissue thickening running anteriorly from the cloaca along the free edge of the posterior mesentery. In cross sections, the thickening has a highly irregular shape and its covering mesothelium is thrown into numerous furrows of varying shape and size (Figure 2C, 4E, F). The mesothelium shows moderate to strong in situ signal, which is somewhat stronger at the antimesenterial side of the rudiment and gradually fades towards the mesenteric attachment (Figure 2C, 4E). It is worth noting that the expression is often weak or missing completely in the cells lining the bottom of the mesothelial folds (Figure 4F).

Moderate to strong *survivin *expression is also seen in the mesothelium and occasionally in the luminal epithelium of the healed posterior tip of the esophageal stump (Figure 2C, 4G) and also in the anterior mesentery that is attached to the tip of the esophageal stump. The hybridization signal is mostly confined to the tall folds of the mesothelium at the free distal edge of the mesentery (Figure 2C, 4H).

**Figure 5:**
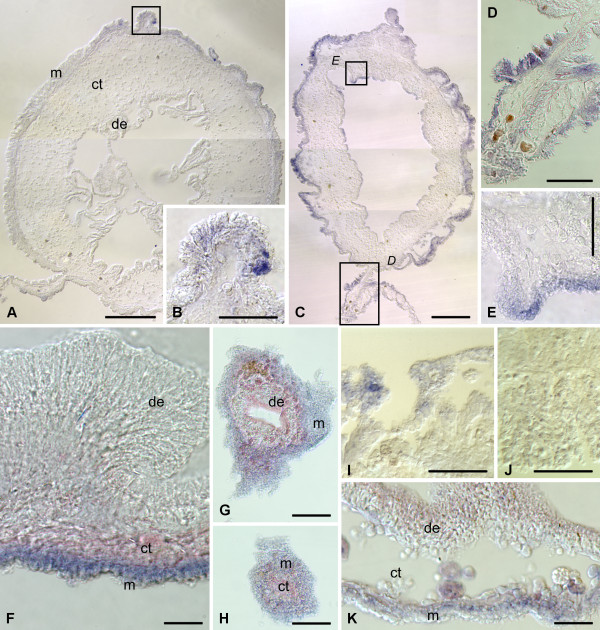
**In situ hybridization**. Expression of *survivin *at advanced stages of gut regeneration (days 12 - 21 after evisceration). **(A) **Section through the posterior rudiment at its attachment to the cloaca on day 12. **(B) **High magnification view of the boxed area on **(A)**. **(C) **Section through the posterior regenerate at a more anterior level relative to **(A)**. **(D) **and **(E) **show high magnification views of the boxed areas on **(C)**, representing the mesenterial attachment and the digestive epithelium, respectively. **(F) **The wall of the esophageal stump on day 12. **(G) **and **(H) **Sequential sections trough the tip of the blindly ended anterior rudiment on day 12. **(I) **and **(J) **The wall of the posterior portion of the regenerated digestive tube on day 21, showing scatted labeled cells in the luminal (digestive) epithelium **(I) **and no labeling in the mesothelium **(J)**. **(K) **The wall of the anterior region of the regenerated gut on day 21, showing expression of *survivin *in the mesothelium. de - digestive (luminal) epithelium; ct - connective tissue layer; m - mesothelium. Scale bars = 100 μm in **(A)**, **(D)**, **(G)**, **(H)**; 50 μm in **(B)**, **(E)**, **(I)**, and **(J)**; 200 μm in **(C); **25 μm in **(F) **and **(K)**.

By day 12-14 after evisceration, the growing luminal epithelia of the esophagus and cloaca invade the connective tissue thickening of the anterior and posterior rudiments, respectively, thereby forming the inner tissue layer of the regenerating gut (Figure 2D). The posterior rudiment shows marked differences in *survivin *expression pattern along its length. In the posterior region, close to the cloaca, the in situ hybridization signal is almost completely absent from the tissues of the regenerate, with the exception of single cells or groups of a few cells in the mesothelium, predominantly on the anti-mesenterial side of the rudiment (Figure 2D, 5A, B). At more anterior levels of the posterior rudiment, *survivin *is expressed with varying intensity over most of the gut mesothelium with some cells showing particularly strong hybridization signal (Figure 2D, 5C). Some expression is also occasionally observed in the coelomic epithelium of the mesentery at its attachment to the posterior gut primordium (Figure 2D, 5C, D). In the luminal epithelium, weak to moderate in situ hybridization signal is often restricted to the apices of the irregularly shaped shallow folds, and this expression is evident mostly in the anti-mesenterial half of the rudiment (Figure 2D, 5C, E).

In the anterior regenerate, *survivin *is broadly expressed at moderate to high levels in the mesothelium of both the esophageal stump and the newly created anterior rudiment, including its very tip (Figure 2D, 5F - H). However, no expression is detected in the luminal epithelium at any level along the anterior primordium.

By days 14 - 21 after evisceration, the continuous lumen is formed in the regenerating gut of eviscerated animals (Figure 2E). In cross sections, the organization of the gut is very similar to that of non-eviscerated animals. The expression pattern of *survivin *in the posterior portion of the newly regenerated gut (second descending region) strongly resembles that of the normal gut (Figure 2E, 5I, J), i.e., there are singly scattered strongly labeled cells in the digestive epithelium (Figure 5I), but no labeling in the mesothelium (Figure 5J). In the anterior portion of the gut, weak to moderate expression is still detected all over the mesothelium (Figure 2E, 5K).

### Spatiotemporal pattern of *mortalin *expression

As revealed by situ hybridization, some regions of the digestive tube, including the esophagus, second descending intestine, and cloaca express *mortalin *under normal conditions (Figure 2A, 3C - E). In the esophagus, *mortalin *is widely expressed in the mesothelium of the gut wall, but within this epithelial layer the hybridization signal is restricted to the apical region, which is known to be occupied predominantly by cell bodies of peritoneocytes [47,49] and where most of mesothelial cell division is observed (Additional File 7), and is absent from the basal region, where myoepithelial cells form the circular musculature of the gut (Figure 3C). In the second descending intestine, moderate to strong expression is observed in the apical region of the coelomic epithelium of the mesentery close to its attachment to the gut. This expression is highly asymmetrical with strong hybridization signal observed only on one side of the mesentery (Figure 2A, 3D). In the cloaca, very rare weakly labeled single cells or groups of a few cells are seen in the luminal epithelium (Figure 3E).

**Figure 6:**
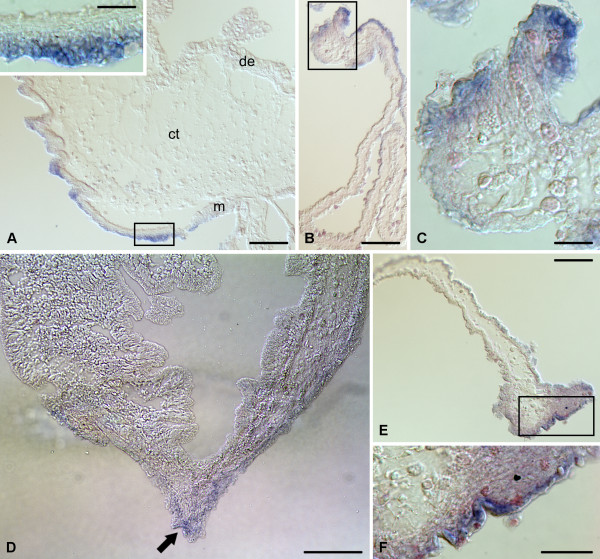
**In situ hybridization**. Expression of *mortalin *in gut tissues on day 2 after evisceration. **(A) **Patchy in situ hybridization signal in the mesothelium of the anterior region of the cloaca. The inset shows a detailed view of the mesothelium corresponding to the boxed area on the main image. **(B) **Low-magnification view of the posterior mesentery. **(C) **A higher magnification of the free distal edge of the posterior mesentery - boxed area in **(B) **- showing strong hybridization signal in some cells of the mesothelium. **(D) **Ventral (anti-mesenterial) region of the esophageal stump showing very weak and restricted in situ hybridization signal (arrow) in the mesothelium. **(E) **Low-magnification view of the anterior mesentery. **(F) **Magnified view of the boxed area in **(E) **showing strongly labeled cells in the mesothelium of the free distal edge of the mesentery. de - digestive (luminal) epithelium; ct - connective tissue layer; m - mesothelium. Scale bars = 100 μm in **(A)**, **(B)**, **(D)**, and **(E)**; 25 μm in **(A inset) **and **(C)**; 50 μm in **(F)**.

On days 2-3 after evisceration, moderate to strong in situ hybridization signal is seen in the mesothelium of the stump of the cloaca. This expression pattern is not continuous, but consists of patches of positive staining interspersed at irregular intervals (Figure 2B, 6A). Clear staining is also observed in the coelomic epithelial cells at the free margin of the posterior mesentery (Figure 2B, 6B, C). The level of *mortalin *expression in the mesothelium of the esophageal stump, when compared with that in the esophagus of non-eviscerated animals (Figure 3C), diminishes significantly, so that it mostly falls below the level of detection by in situ hybridization technique and only patches of very weak staining are occasionally observed in some areas of the mesothelium (Figure 2B, 6D). In contrast, the region of the mesentery that lies just posterior to the tip of the esophageal stump shows very intense labeling in some cells of the coelomic epithelium at its free edge (Figure 2B, 6E, F).

**Figure 7:**
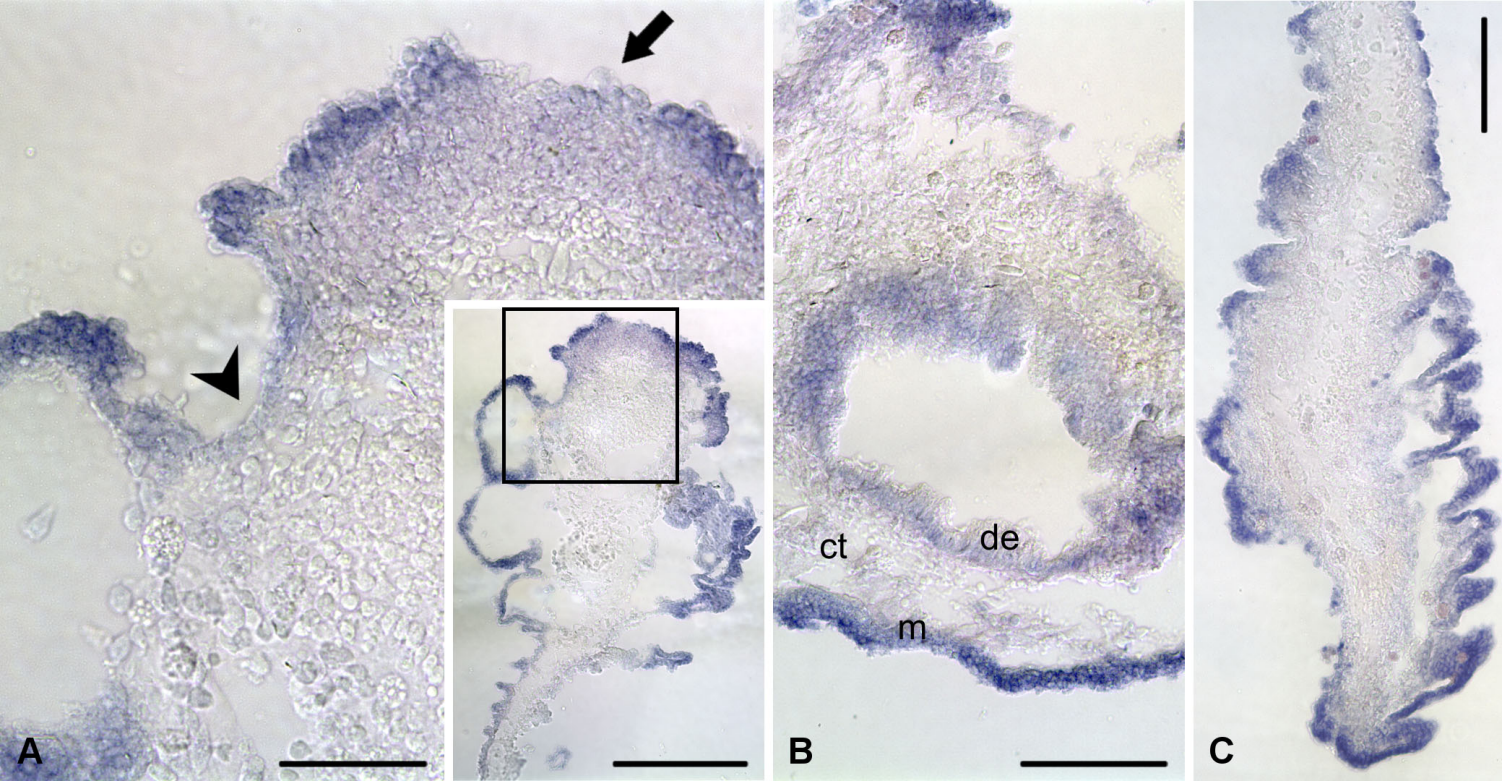
**In situ hybridization**. Expression of *mortalin *in gut tissues on day 6 after evisceration. **(A) **In situ hybridization signal in the mesothelium of the posterior rudiment. Note that the signal is weak or completely absent from the anti-mesenterial region of the rudiment (arrow) and from the bottom of mesothelial furrows (arrowhead). The inset shows a low-magnification view of the posterior rudiment with the boxed area corresponding to the main image. **(B) **The posterior tip of the esophageal stump. Note strong in situ hybridization signal in the mesothelium and moderate signal in the luminal (digestive) epithelium. **(C) **Strong expression of *mortalin *in the mesothelium of the anterior mesentery. de - digestive (luminal) epithelium; ct - connective tissue layer; m - mesothelium. Scale bars = 50 μm in **(A)**; 200 μm in **(A inset)**; 100 μm in **(B) **and **(C)**.

On days 6 - 7, *mortalin *is widely expressed in the mesothelium of the irregularly shaped posterior rudiment (Figure 2C, 7A). However, the expression is often weak or completely undetectable in the anti-mesenterial region of the rudiment and also at the bottom of the mesothelial furrows. In the stump of the esophagus and in the mesentery running posteriorly from the stump tip, *mortalin *is expressed all over the mesothelium at moderate to high levels (Figure 2C, 7B, C). At the sealed tip of the stump, where the lumen ends blindly, most of the cells of the digestive epithelium also show weak to moderate hybridization signal (Figure 7B).

On days 12-14 after evisceration, *mortalin *is strongly expressed all along the mesothelium of the posterior regenerate, including the anti-mesenterial region (Figure 2D, 8A). There are some local variations in the intensity of the signal between adjacent regions of the mesothelium in cross-sections, but those variations does not form any regular pattern. The localization of the hybridization signal shows no considerable differences along the rudiment either. No labeling is detected in the luminal epithelium of the posterior gut primordium.

**Figure 8:**
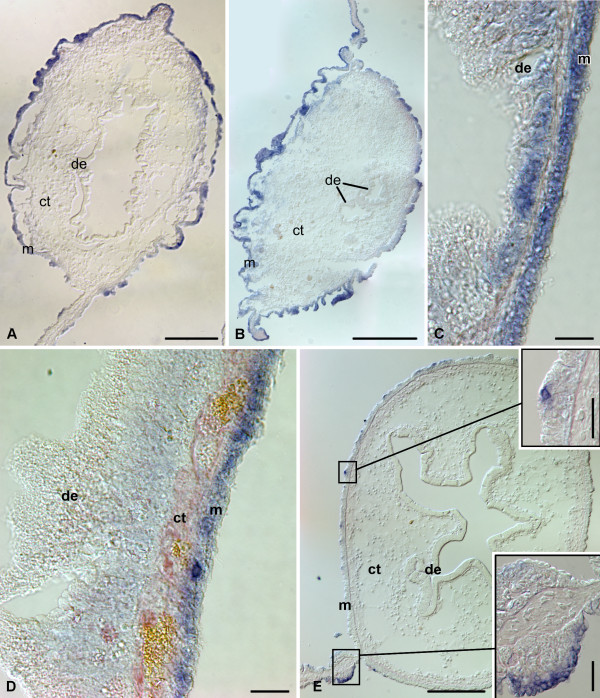
**In situ hybridization**. Expression of *mortalin *at advanced stages (days 12 - 21) of gut regeneration. **(A) **Posterior rudiment on day 12 after autotomy. **(B) **Growing posterior tip of the anterior rudiment on day 12 after evisceration. **(C) **The wall of the esophageal stump on day 12 after evisceration. **(D) **The wall of the newly regenerated posterior regions of the esophagus on day 21 after evisceration. **(E) **The second descending part of the newly regenerated intestine on day 21 after evisceration. The inserts show higher magnification view of the asymmetrical expression of *mortalin *in the mesothelium of the mesentery attachment and also strongly labeled singly scattered cells in other regions of the mesothelium. de - digestive (luminal) epithelium; ct - connective tissue layer; m - mesothelium. Scale bars = 200 μm in **(A) **and **(E)**; 500 μm in **(B)**; 25 μm in **(C)**, **(D) **and **(E insets)**.

The distribution of the *mortalin *in situ hybridization signal in the anterior rudiment is similar to that in the posterior rudiment, i.e., moderate to strong expression is seen mostly in the mesothelium (Figure 2D, 8B) with little or no variation between different regions in cross-sections and along the length of the primordium. Unlike at the previous stage, no expression is seen in the luminal epithelium at the growing tip of the anterior regenerate (Figure 8B), but *mortalin *transcripts are occasionally detected in the groups of cells in the luminal epithelium of the esophageal stump (Figure 8C).

On days 14 - 21, the spatial expression pattern of *mortalin *in the tissues of the newly regenerated digestive tube does not differ much from that of the non-eviscerated individuals (Figure 2E, 8D, E). For instance, moderate to strong hybridization signal is seen all over the mesothelium of the anterior region of the regenerate (esophagus) (Figure 8D) and on one of the sides of the mesentery attachment to the posterior regenerate (second descending part of the intestine) (Figure 8E). The only difference from the expression pattern in the intact gut is the presence of single scattered intensely labeled cells in the mesothelium at various levels along the regenerate. No significant hybridization signal is detected in the digestive (luminal) epithelium at this stage.

### Qualitative assessment of transcript abundance

The overall relative abundance of the *survivin *and *mortalin *transcripts in the tissue samples was assessed by real-time qualitative PCR. All data were compared to the normal gut and shown as fold change.

**Figure 9:**
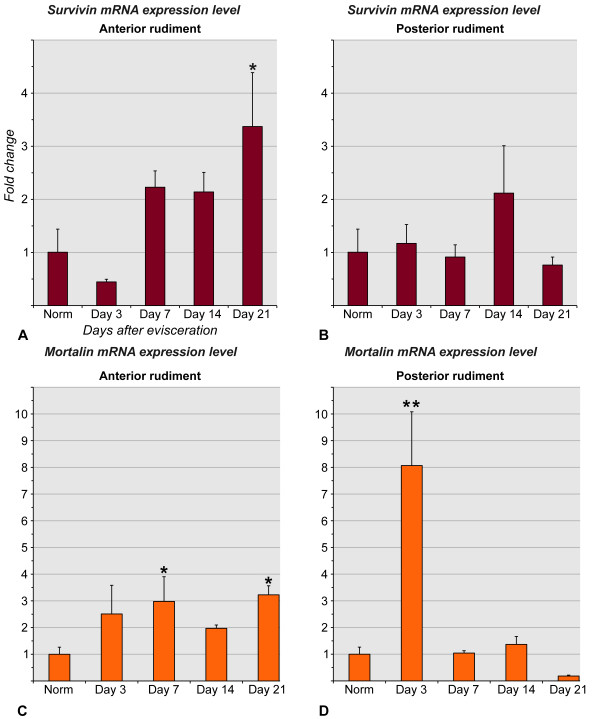
**Real-time RT-qPCR**. Overall abundance of *survivin *and *mortalin *transcripts in the regenerating digestive tube. **(A) **and **(B) ***Survivin *expression in the anterior and posterior regenerates, respectively. **(C) **and **(D) ***Mortalin *expression in the anterior and posterior regenerates, respectively. Transcript abundance is expressed as x-fold relative to the normal gut. Results are represented as mean ± standard error. *P < 0.05, **P < 0.01

Both *survivin *(Figure 9A, B) and *mortalin *(Figure 9C, D) showed different temporal expression profiles in the anterior and posterior regenerates of the digestive tube. No significant difference in the overall *survivin *expression was found between the posterior regenerate at any stage of re-growth and the intact digestive tube (p ≥ 0.2269) (Figure 9B), whereas the anterior regenerate shows a marginally insignificant increase in *survivin *mRNA level on days 7 (p = 0.067) and 14 (p = 0.073), which becomes a significant (p = 0.037) three-fold increase by day 21 after evisceration (Figure 9A).

Real-time RT-PCR analysis of *mortalin *transcript abundance in the anterior rudiment reveals two peaks of roughly 3-fold up-regulation, one on day 7 (p = 0.036) and another one on day 21 (p = 0.019) (Figure 9C), while, in the posterior rudiment, the overall *mortalin *expression level shows a highly significant increase as early as on day 3 after evisceration (p = 0.003) and then returns to the approximately normal level on days 7 to 21 (Figure 9D).

### Apoptosis

**Figure 10:**
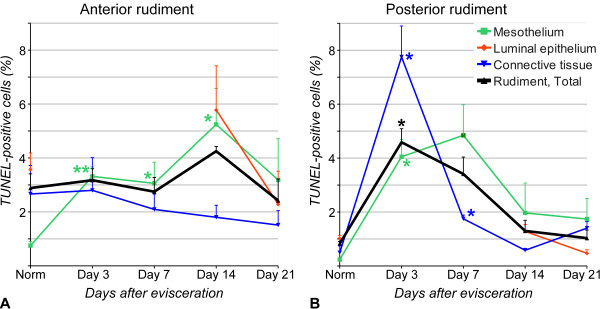
**TUNEL assay**. Percentage of apoptotic cells in tissue layers of the normal and regenerating digestive tube. **(A) **Cell death in the anterior regenerate. **(B) **Apoptosis in the posterior regenerate. Results are represented as mean ± standard error. *P < 0.05, **P < 0.01

**Figure 11:**
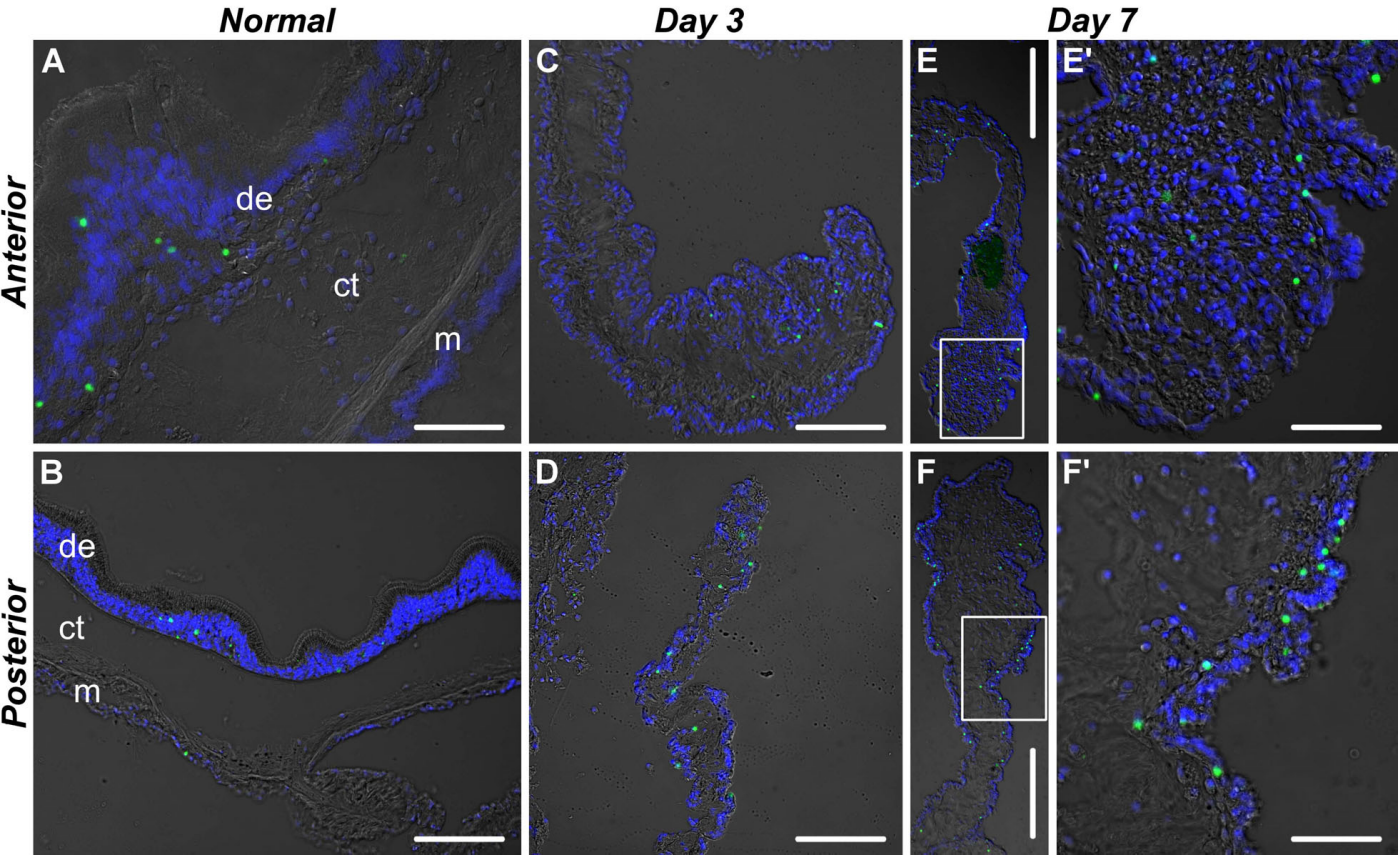
**Representative micrographs of the distribution of TUNEL-positive cells (green) in the normal gut and in the early regenerates (days 3 - 7)**. **(A) **Wall of the esophagus in a non-eviscerated animal. **(B) **Wall of the second descending intestine in a non-eviscerated animal. **(C) **and **(D) **The anterior and posterior regenerates, respectively, on day 3. **(E) **and **(F) **General view of the anterior and posterior regenerates, respectively, on day 7. **(E') **and **(F') **Higher magnification of the boxed areas on **(E) **and **(F)**, respectively. de - digestive (luminal) epithelium; ct - connective tissue layer; m - mesothelium. TUNEL-positive cells are green; nuclei were stained with DAPI and are shown in blue. Scale bars = 50 μm in **(A)**, **(E')**, and **(F')**; 100 μm in **(B) **- **(D)**; 200 μm in **(E) **and **(F)**.

**Figure 12:**
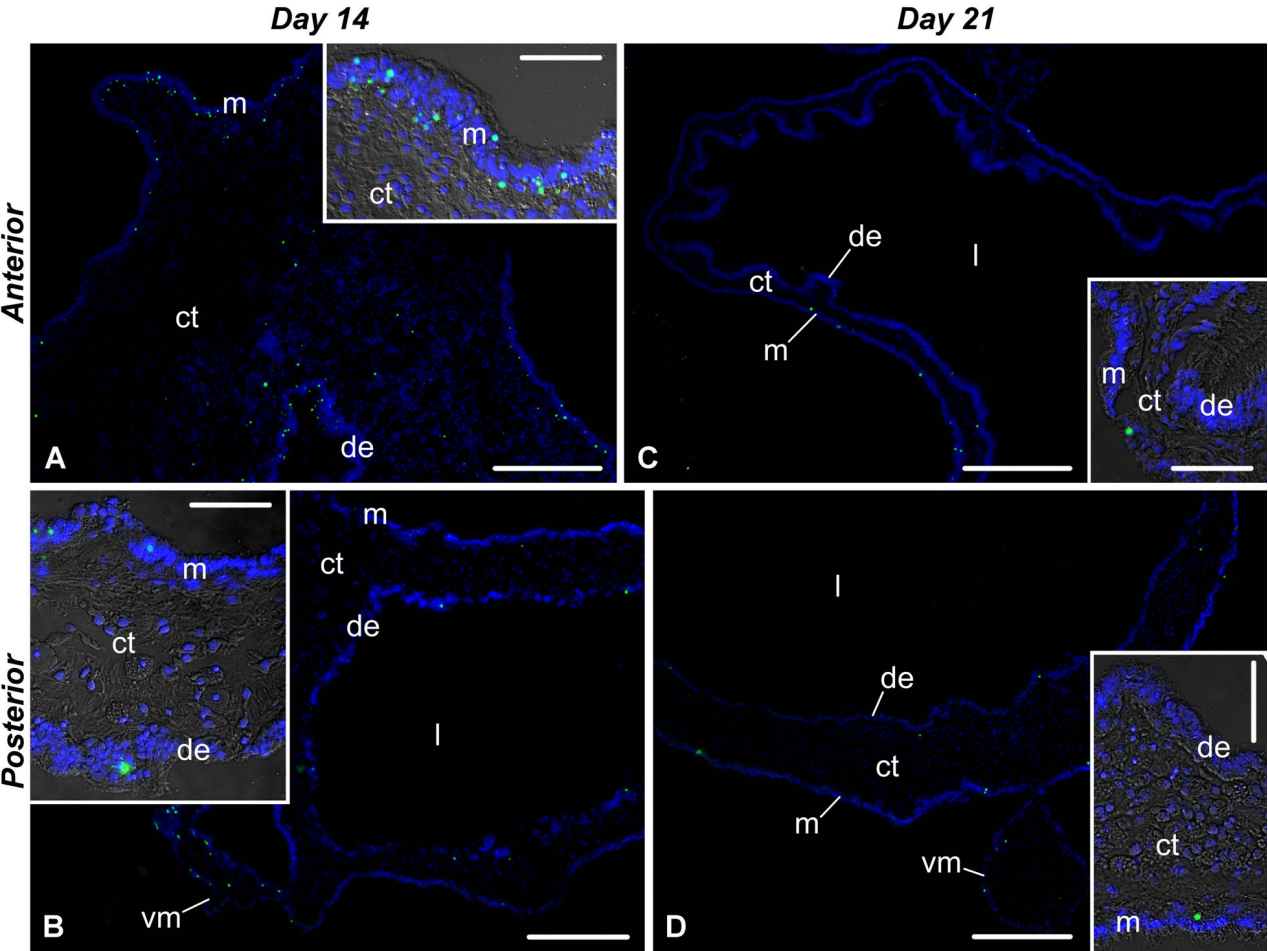
**Representative micrographs of the distribution of TUNEL-positive cells during the late phase (days 14 and 21) of visceral regeneration**. **(A) **and **(B) **The anterior and posterior regenerates, respectively on day 14. **(C) **and **(D) **The newly regenerated posterior region of the esophagus and the second descending intestine on day 21 after evisceration. Insets show higher magnification views of the gut wall. de - digestive (luminal) epithelium; ct - connective tissue layer; l - gut lumen; m - mesothelium; vm - visceral mesentery. TUNEL-positive cells are green; nuclei were stained with DAPI and are shown in blue. Scale bars = 200 μm in **(A) **- **(D)**; 50 μm in all **insets**.

Since both survivin and mortalin are known to act as anti-apoptotic proteins, we performed TUNEL assay to examine the extent of programmed cell death in the normal and regenerating digestive tube. Figure 10 shows the diagrams of the temporal changes in the percentage of TUNEL-positive cells in the regenerating digestive tube, and Figure 11 and 12 are representative micrographs used in cell counting assays.

As could be expected, some cell death occurs even in the tissues of the non-eviscerated digestive tube, the dying cells being mostly restricted to the luminal epithelium (Figure 10, 11A, B). The apoptotic cells are significantly (Welch's t-test, p = 0.03) more abundant in the anterior regions (esophagus) of the digestive tube (2.88 ± 0.53% of the total cell number), than in the posterior part (0.83 ± 0.08%). As early as on day 3 after evisceration, the mesothelium of both the anterior and posterior regenerates shows a significant increase (4-fold and almost 17-fold, respectively) in percentage of TUNEL-positive cells (with the corresponding p-values of 0.009 and 0.03, respectively) (Figure 10, 11C, D). In the mesothelium of the anterior regenerate, this elevated level of cells death persists until day 14 and then declines at later stages (Figure 10A). In the mesothelium of the posterior rudiment, the increase in percentage of TUNEL-positive cells remains almost significant until day 7 (p = 0.056), before declining later on (Figure 10).

Cells in the connective tissue of the anterior and posterior rudiments respond differently to injury. In the anterior rudiment, no significant changes in percentage of TUNEL-positive cells were observed (Welch's ANOVA, F(4, 5.9) = 0.32, p = 0.86) at any of the regeneration stages studied, whereas the connective tissue layer of the posterior rudiment shows a sharp increase in cell death on day 3 after autotomy (p = 0.02), which is followed by a rapid decline, and, by day 14 of regeneration, the percentage of TUNEL-positive cells resumes its normal values (Figure 10). As the new luminal epithelium develops on days 14 - 21 after evisceration, it does not show any stage-dependent variation in the overall rate of cell death (anterior rudiment: F(2, 3.5) = 1.22, p = 0.4; posterior rudiment: F(2, 3.6) = 6.24, p = 0.07, Welch's ANOVA) (Figure 10, 12).

### Multiple labeling

**Figure 13:**
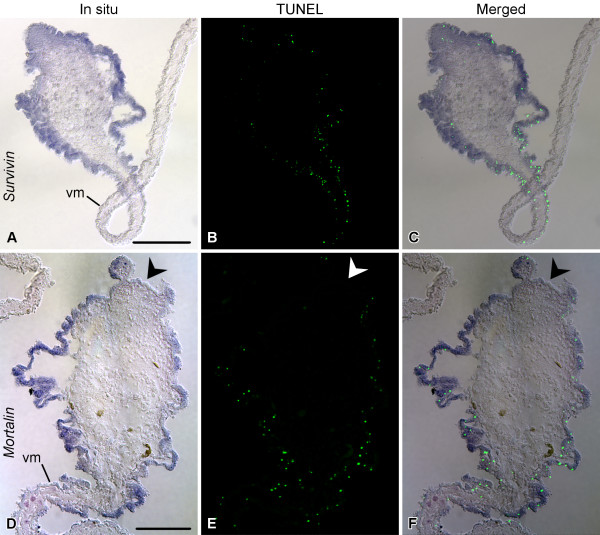
**Double labeling with riboprobes for *survivin *and *mortalin *(blue) and TUNEL assay (green) on the posterior regenerate on day 7**. **(A) **- **(C) ***Survivin *riboprobe and TUNEL assay. **(D) **- **(F) ***Mortalin *riboprobe and TUNEL assay. vm - ventral mesentery. Arrowhead on **(D) **- **(F) **marks the anti-mesenterial region of the rudiment, where *mortalin *transcript are absent. Note a negative correlation between the localization of *survivin *in situ hybridization signal and the density of the TUNEL-positive cells **(A) **- **(C)**. Scale bars = 100 μm.

**Figure 14:**
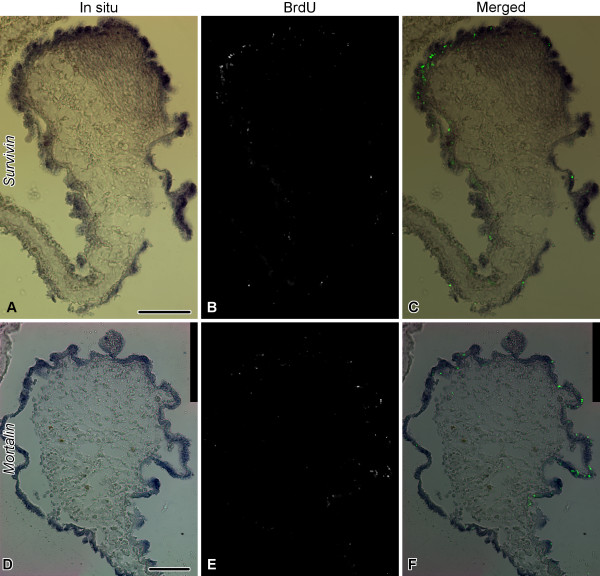
**Double labeling with riboprobes for *survivin *and *mortalin *(blue) and BrdU immunocytochemistry (green) on the posterior regenerate on day 7**. **(A) **- **(C) ***Survivin *riboprobe and BrdU immunohistochemstry. **(D) **- **(F) ***Mortalin *riboprobe and BrdU immunohistochemistry. Note that BrdU-incorporating cells are mostly distributed within the expression domains of *survivin ***(A) **- **(C) **and *mortalin ***(D) **- **(F)**. Scale bars = 100 μm.

To obtain some insight into possible function(s) of *survivin *and *mortalin *in sea cucumber gut regeneration, we performed double labeling experiments by subjecting the samples of the posterior gut rudiment at the stage of 7 days after evisceration (extensive expression of both genes, elevated levels of both cell death and proliferation [5]) to in situ hybridization followed by either TUNEL assay or BrdU immunohistochemistry. In the lateral and anti-mesenterial regions of the regenerate, where *survivin *transcripts are most abundant, TUNEL-positive cells are rare. However, the region where the newly developing gut attaches to the mesentery is characterized both by a weaker *survivin *mRNA hybridization signal and an increased abundance of apoptotic cells in the mesothelium (Figure 13A-C). Therefore, there is a negative correlation between the survivin level and the extent of cell death in the coelomic epithelial cells. The spatial relationship between *mortalin *expression and the cell death is less straightforward, because, although *mortalin *transcripts are seen in the lateral regions of the regenerate, where cell death is less extensive than in the mesenterial attachment, they are often absent from the mesothelium covering the anti-mesenterial region of the rudiment, where the apoptotic cells are scarcely seen (Figure 13D - F). Combined in situ hybridization and BrdU immunohistochemistry shows that the localization of the cell division in the regenerating mesothelium largely coincides with the expression domains of *survivin *and *mortalin *(Figure 14).

## Discussion

Cell death and cell division are the two fundamental processes that create tissue homeostasis. The ability to tightly control them is of vital importance for any multicellular organism. Although one can intuitively perceive that regeneration will certainly shift the balance in favor of cell proliferation, induction of apoptosisis is nevertheless known to be equally important for successful regeneration, and, moreover, can be absolutely required to trigger tissue repair [37,38]. Visceral regeneration in the sea cucumber *H. glaberrima *involves both extensive cell proliferation, as was documented earlier [5], and a transient increase in programmed cell death in the regrowing tissues, as we have shown here. The present study examines the expression pattern of *survivin *and *mortalin*, known to be involved in both mitosis and apoptosis in various animal taxa, in the normal and regenerating gut of the sea cucumber.

Both genes show certain basal levels of expression in the digestive tube of non-eviscerated sea cucumber individuals (Figure 2A, 9). Survivin protein is believed to be absent from most of the adult tissues of vertebrates. Notable known exceptions include organs with high rate of physiological cell turnover, such as thymus and gastric mucosa [13,27,28]. In our study, we detected *survivin *transcripts in single cells widely scattered throughout the luminal (digestive) epithelium of the sea cucumber. Unlike *survivin*, *mortalin *is almost entirely absent from the luminal epithelium of the normal gut with the exception of vary rare cells in the digestive epithelium of the cloaca. However, *mortalin *is widely expressed in the mesothelium (coelomic epithelium) of the esophagus and also shows an interesting asymmetric expression pattern at the attachment of the mesentery to the posterior region of the intestine. Functional significance of the expression of the two genes in the normal digestive tube is not yet clear, but since both epithelia of the holothurian gut are known to slowly self-renew [49] (Additional File 7), involvement in cell turnover could be a possible explanation.

Visceral autotomy (evisceration) in sea cucumbers triggers a series of developmental events, such as apoptosis, cell division, migration of individual cells and epithelial sheets, connective tissue remodeling, de-differentiation, trans-differentiation, and re-differentiation [5,8,48,50]. These events, although partially overlapping in time and space, unfold in a certain temporal order to eventually result in the successful regeneration of the digestive tube. Likewise, both *survivin *and *mortalin *show time-dependent changes in their expression in the regenerating digestive tube of the sea cucumber. These changes do not necessarily manifest themselves in increased or decreased numeric values of the overall relative abundance of mRNA transcripts, but can involve mostly changes in the spatial distribution of the transcripts instead. For instance, although the overall quantity of *survivin *transcripts in the posterior gut regenerate of *H. glaberrima *does not change significantly (relative to the non-eviscerated gut), in situ hybridization shows marked spatial alterations of the expression pattern as regeneration progresses.

In situ hybridization revealed that both *survivin *and *mortalin *are much more abundantly expressed in the mesothelium of the regenerating gut, than in the luminal epithelium, and that the two genes are completely absent from the cells of the connective tissue layer. The visceral mesothelium of echinoderms shows a complex histological organization [8,47,49]. It is mostly made up of specialized peritoneocytes and myoepithelial cells, which are assembled in a highly organized architecture, and also contains a basiepithelial nerve plexus. In spite of this high level of histological complexity, the mesothelium of sea cucumbers is known to possess an extraordinary histogenetic potential even in adult animals. Shortly after evisceration, the mesothelia of the mesentery and the remaining portions of the digestive tube (stumps) undergo drastic de-differentiation, which transforms the highly specialized tissue into a layer of greatly simplified peritoneal and myoepithelial cells, which lose their characteristic properties, such as long basal processes and myofilaments, respectively [6-8,51]. In this dedifferentiated condition, the mesothelium undergoes extensive cell division and expands to accommodate the connective tissue swelling, which is being developed along the free edge of the mesentery. The combination of the connective tissue thickening with the surrounding dedifferentiated mesothelium constitutes the early regenerate of the digestive tube. As regeneration progresses, the mesothelium gradually resumes its normal organization, i.e., undergoes re-differentiation.

In the regenerating digestive tube of *H. glaberrima*, the most extensive expression of *survivin *and *mortalin *in the mesothelium occurs on days 3 through 14 after evisceration. This is exactly the time when the mesothelium shows a significant increase in cell death (this study) and cell division [5], and undergoes the dedifferentiation - expansion - redifferentiation cycle [5,7,8]. Therefore, since the activation of *survivin *and *mortalin *expression coincides in time with the major morphogenic processes, the two genes are likely to be involved somehow in the remodeling of the gut mesothelium during regeneration. For instance, the peak in cell proliferation in the regenerating gut mesothelium of *H*. *glaberrima *occurs on days 7 though 14 after evisceration [5], and since both genes are widely expressed in the same regions where proliferation occurs (this study) at these stages, it is possible that they are involved in regulation of cell division.

There is a discrepancy in the literature on the anti-apoptotic function of *survivin *in different regenerating tissues. In human and rodent liver regeneration, *survivin *up-regulation was related to cell proliferation, but not to apoptosis inhibition [18,19]. On the other hand, a role for *survivin *in suppression of the programmed cell death was demonstrated in traumatic brain injury in rats [52]. Combined in situ hybridization and TUNEL labeling of the regenerating gut of *H. glaberrima *showed that, although *survivin *expression and the increase in cell death rate occurred concomitantly in the mesothelium, the strongest *survivin *hybridization signal and the highest abundance of apoptotic cells were mostly localized to different territories (the basal plus lateral surfaces of the rudiment and the mesenterial attachment, respectively), suggesting an anti-apoptotic role for *survivin *in the regenerating coelomic epithelium of the holothurian gut. No such clear relationship exists between the cell death and *mortalin *expression, which can be explained by a variety of other functions that the mortalin protein is known to perform [14].

In terms of cell sources of regeneration, there are two major groups of events: those that involve some kind of undifferentiated reserve/stem cells (such as neoblasts in planarians) [53] and those that rely on the plasticity of the existing differentiated cells (as in case of mammalian pancreatic beta-cells and liver regeneration after acute injury) [54,55]. Previous electron microscopy studies [6-8] clearly demonstrated that both the luminal epithelium and the mesothelium of the sea cucumber digestive tube regenerate through induction of extensive proliferation of the differentiated cells resulting in expansion of the tissue layers of the gut stumps into the regenerate. The peritoneal and myoepithelial cells of the mesothelium undergo drastic dedifferentiation by losing their characteristic features and enter the cell cycle, but remain nevertheless connected to each other by intercellular junctions within the epithelial sheet. The present study shows that the mesothelium in this dedifferentiated condition expresses *mortalin *and *survivin*. In this regard, it is important to note that the expression of these two genes is known to be associated with stem cells. *Mortalin*, for instance, is constitutively expressed by planarian neoblasts and its knockdown results in inability to regenerate and maintain normal cell turnover [20]. *Survivin *is known to be expressed in stem cells of a variety of tissues undergoing cell turnover [24,56], where it is though to contribute to stem cell maintenance and protection from cell death. Therefore, the results of the present study combined with the data obtained earlier, suggest that, although the mesothelium of the sea cucumber gut is devoid of resident stem cells, most of the mesothelial cells themselves temporarily acquire some stem cell properties through reversible dedifferentiation. Those properties include the absence of specialized cytoplasmic features, ability to go through cell divisions, and expression of *survivin *and *mortalin*. It is worth mentioning here that in vitro studies of the cells derived from sea cucumber visceral regenerates showed that only the cells obtained during the phase of extensive dedifferentiation and proliferation, were capable of sustained growth in culture [9].

It is not clear why the transcripts of both *survivin *and *mortalin *are much less abundant in the regenerating luminal epithelium, than in the mesothelium. The luminal epithelium *H. glaberrima*, as in other members of the order Aspidochirota, also regenerates via proliferation of the enterocytes that remain in the esophageal and cloacal stumps after evisceration [5,7,9], i.e. employs the same basic mechanisms, as the mesothelium. The obvious explanation is that regeneration of the luminal epithelium may employ additional pathways, besides inducing extensive expression of *survivin *and *mortalin*, to coordinate cell death and/or proliferation. This conclusion is in line with findings that, contrary to a previous belief, the survivin protein is not absolutely required to prevent cell death during mitosis [57].

The differences in regeneration mechanisms of the same tissue between different species or between different developmental stages of the same animal are not uncommon and are not surprising. However, visceral regeneration in holothurians provides an intriguing example of how different cellular and/or molecular mechanisms can be employed simultaneously in the same organ of the same individual. One of the most extreme studied cases is gut regeneration in a dendrochirotid holothurian *Eupentacta fraudatrix *[8]. In this species, the luminal epithelium in the posterior gut rudiment, as could be expected, develops from the luminal epithelium of the cloacal stump, whereas the luminal epithelium of the anterior regenerate develops from the mesodermally derived cells of the mesothelium. In spite of different origin, the anterior and posterior luminal epithelia are indistinguishable from each other in histological and ultrastructural organization once regeneration is completed. In *H. glaberrima*, the species used in the present study, the differences in mechanisms between the anterior and posterior rudiments are not as prominent, but still are present (Figure 2, 9, 10). They include different timing of *survivin *and *mortalin *expression peaks, somewhat different pattern of spatial distribution of the transcripts of the two genes, as well as some differences in the programmed cell death dynamics, particularly in the connective tissue layer. It is not clear yet whether the differences in regeneration mechanisms between the anterior and posterior regenerates are related to the oral-aboral polarity of the animal, reflect the evolutionary history of the regenerative mechanisms, or have any adaptive significance.

## Conclusions

All developmental events (broadly defined) including embryogenesis, postnatal cell turnover, tumor formation, and regeneration rely on the balance between cell division and cell death. Understanding the basic mechanisms that regulate these two processes is not only of great academic interest but also holds promise for medical advances. The present study examines the expression pattern of *survivin *and *mortalin*, two genes known to be involved in regulation of both cell division and apoptosis, in the regenerating viscera of the sea cucumber *Holothuria glaberrima*. In response to injury, both genes show changes in the spatial distribution of the transcripts and/or in the overall abundance of the transcripts in the gut regenerates. Although the two genes show some expression in the regenerating luminal epithelium (at certain stages, in certain regions), the most extensive expression is seen in the mesothelium (the outer layer of the gut) at days 6 through 14, the stage, at which the mesothelial cells are known to be dedifferentiated and engaged in extensive proliferation [5,7-9]. Our data also show elevated levels of cell death in the regenerating mesothelium. Double labeling experiments suggest that both genes are likely to support cell proliferation in the regenerating gut, while *survivin *might also be involved in apoptosis suppression. It also cannot be ruled out that the two genes play some other additional functions in the regenerating tissues.

The very fact that *survivin *and *mortalin *are expressed in the sea cucumber digestive tube raises an interesting question. Since both genes are known to be involved in carcinogenesis [14,58], why is it that tumor formation has never been reported in studies of visceral regeneration in holothurians or documented in animals captured in the wild? In metazoans (multicellular organisms) with a relatively long life span, the ability to replace worn-out cells under normal conditions and/or replenish the cell mass lost to injury strongly correlates with the presence of potent tumor suppression mechanisms that keep the rate of cell division within secure limits to match the interests of the organism as a whole [59]. Sea cucumbers are characterized by a relatively long life span, estimated at about four to ten years [60], they constantly renew cells in their adult tissues, including the digestive tube [49] and, most interestingly, they can quickly regrow most of their tissues after traumatic injury, autotomy, or seasonal atrophy [1,2] and regenerate the same structure multiple times over their lifetime. Therefore, sea cucumbers, and echinoderms in general, must have evolved a particularly strong set of anti-tumor mechanisms, further studies of which could improve our understanding of relationships between embryogenesis, cancer and regeneration, might help us to devise more effective cancer treatment strategies.

## List of abbreviations

BIR: baculovirus inhibition of apoptosis protein repeat; BIRC5: baculoviral IAP repeat-containing protein 5; BrdU: 5-bromo-2-deoxyuridine; DIG: digoxigenin; Grp75: 75 kDa glucose-regulated protein; Hspa9: heat shock 70 kDa protein 9; IAP: inhibitor of apoptosis proteins; ORF: open reading frame; PBP74: peptide-binding protein 74; RT-qPCR: reverse transcription: quantitative polymerase chain reaction; TUNEL: deoxynucleotidyl transferase-mediated dUTP nick end labeling.

## Authors' contributions

VSM, ORZ, CRC, and JGA conceived the study and interpreted the results. CRC and JGA performed the EST data analysis and identified the genes. VSM and ORZ carried out the experimental procedures and analyzed the data. VSM drafted the manuscript. VSM, ORZ, and JGA finalized the manuscript. All authors read and approved the final version of the manuscript.

## Supplementary Material

Additional file 1 **PCR primers used in the present study**. Click here for file

Additional file 2 **Alignment of survivin protein sequences from *H. glaberrima *and other deuterostome species**. Conservative residues are shaded in blue. The BIR domain is framed in red. Red asterisks mark the conserved residues, which form a zinc finger that stabilizers the structure of the BIR domain [22]. For the accession numbers of the sequences used in the alignment, see Additional File 3. Click here for file

Additional file 3 **The overall similarity between the deduced amino acid sequence of *H*. *glaberrima *survivin and survivin orthologs of other deuterostomes**. Click here for file

Additional file 4 **Alignment of the ATPase domain of mortalin from *H. glaberrima *and other deuterostome species**. Conservative residues are shaded in blue. For the accession numbers of the sequences used in the alignment, see Additional File 6. Click here for file

Additional file 5 **Alignment of the substrate-binding of mortalin sequences from *H. glaberrima *and other deuterostome species**. Conservative residues are shaded in blue. For the accession numbers of the sequences used in the alignment, see Additional File 6. Click here for file

Additional file 6 **The overall similarity between the deduced amino acid sequence of *H*. *glaberrima *mortalin and mortalin orthologs of some other deuterostomes**. Click here for file

Additional file 7 **BrdU-positive cells (green) in the apical region of the mesothelium of the esophagus in a non-eviscerated individual of *H. glaberrima***. Arrowheads show BrdU-positive cells. Nuclei were stained with propidium iodide and are shown in blue. ct - connective tissue layer; de - digestive (luminal) epithelium; m - mesothelium. Scale bar = 25 μm. Click here for file
