# Supplementary material for: Visceral regeneration in a sea cucumber involves extensive expression of survivin and mortalin homologs in the mesothelium
Source: BMC Dev Biol. 2010 Nov 29;10:117. doi: 10.1186/1471-213X-10-117 (PMC3013081; doi:10.1186/1471-213X-10-117)
Supplement: Additional file 1 — PCR primers used in the present study. [file 1471-213X-10-117-S1.PDF]

Additional File 1.

PCR primers used in the present study

Target gene	Primers	Sequence (5' → 3')	Application
Survivin	Surv-qPCR-F	TTACCACTGCCCAACAGACA	Real-time qPCR
	Surv-qPCR-R	TCCTCCCATGGATCATCACT	Real-time qPCR
	Surv-F-templ	TATAACCCTACAAATCGAA	Pre-template synthesis
	Surv-R-templ	AGCACCTCACAACATCTGA	Pre-template synthesis
	T7-Surv-F-templ	TAATACGACTCACTATAGGGTATAACCCTACAAATCGAA	Template generation
	T7-Surv-R-templ	TAATACGACTCACTATAGGGAGCACCTCACAACATCTGA	Template generation
Mortalin	Mort-qPCR-F	GGCATCTTCAGCCCTCCAA	Real-time qPCR<br>Pre-template synthesis
	Mort-qPCR-R	GCAGCCTGTCTGTTCAGTTCTTG	Real-time qPCR
	Mort-R-templ	TGGTTTGCTTGCCATTTGA	Pre-template synthesis
	T7-Mort-F-templ	TAATACGACTCACTATAGGGGGCATCTTCAGCCCTCCAA	Template generation
	T7-Mort-R-templ	TAATACGACTCACTATAGGGTGGTTTGCTTGCCATTTGA	Template generation

Target gene	Primers	Sequence (5' → 3')	Application
**NADH dehydrogenase subunit 5**	NADH-qPCR-F	CAATGGTTGTTGCTGGAGTCTTT	Real-time qPCR
	NADH-qPCR-R	CGCAGAAGTAGCCGCGAATAT	Real-time qPCR
