# Supplementary figures and images for: Visceral regeneration in a sea cucumber involves extensive expression of survivin and mortalin homologs in the mesothelium
Source: BMC Dev Biol. 2010 Nov 29;10:117. doi: 10.1186/1471-213X-10-117 (PMC3013081; doi:10.1186/1471-213X-10-117)

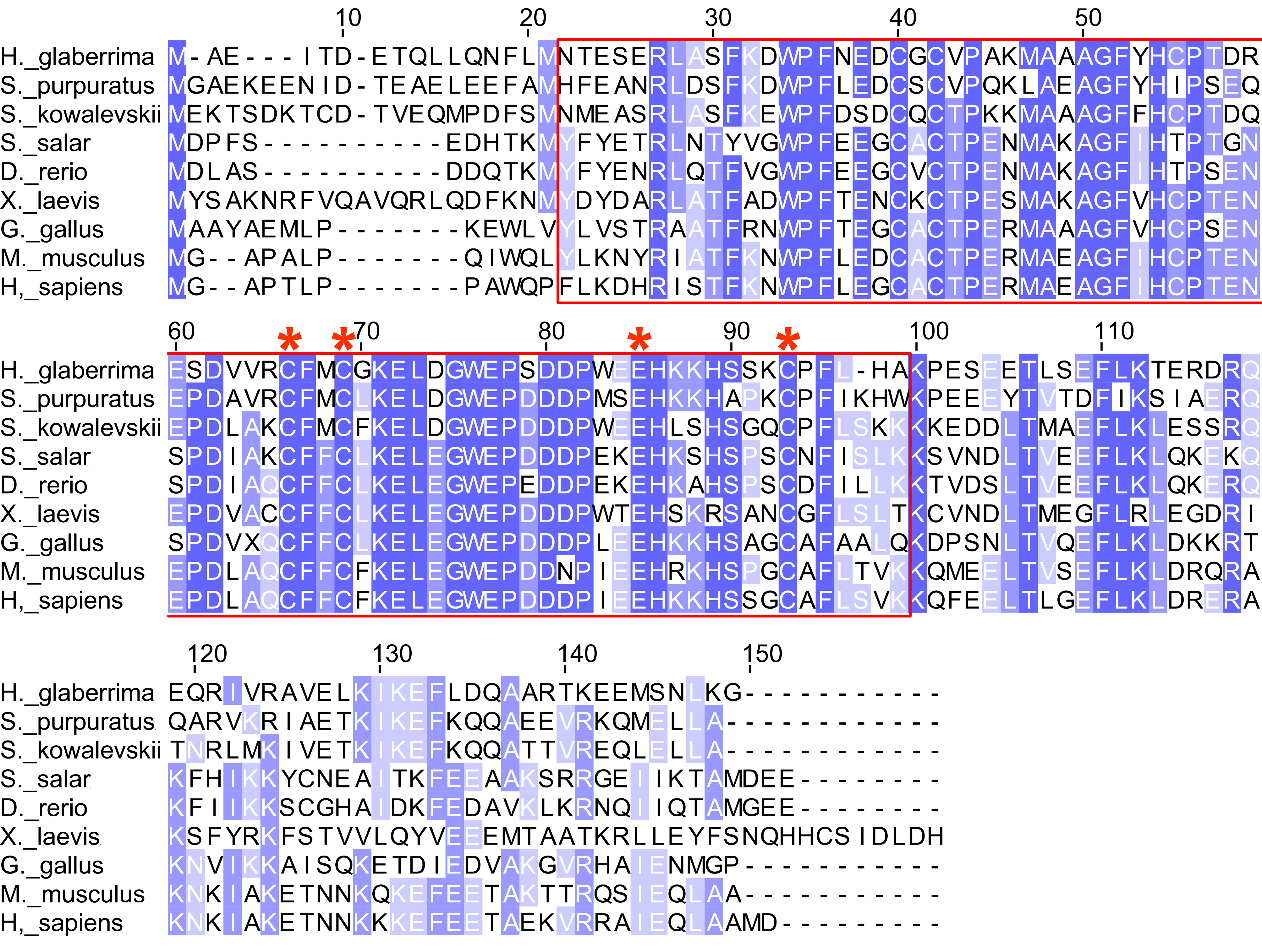

Supplement: Additional file 2 — Alignment of survivin protein sequences from H. glaberrima and other deuterostome species. Conservative residues are shaded in blue. The BIR domain is framed in red. Red asterisks mark the conserved residues, which form a zinc finger that stabilizers the structure of the BIR domain [22]. For the accession numbers of the sequences used in the alignment, see Additional File 3. [file 1471-213X-10-117-S2.TIFF]

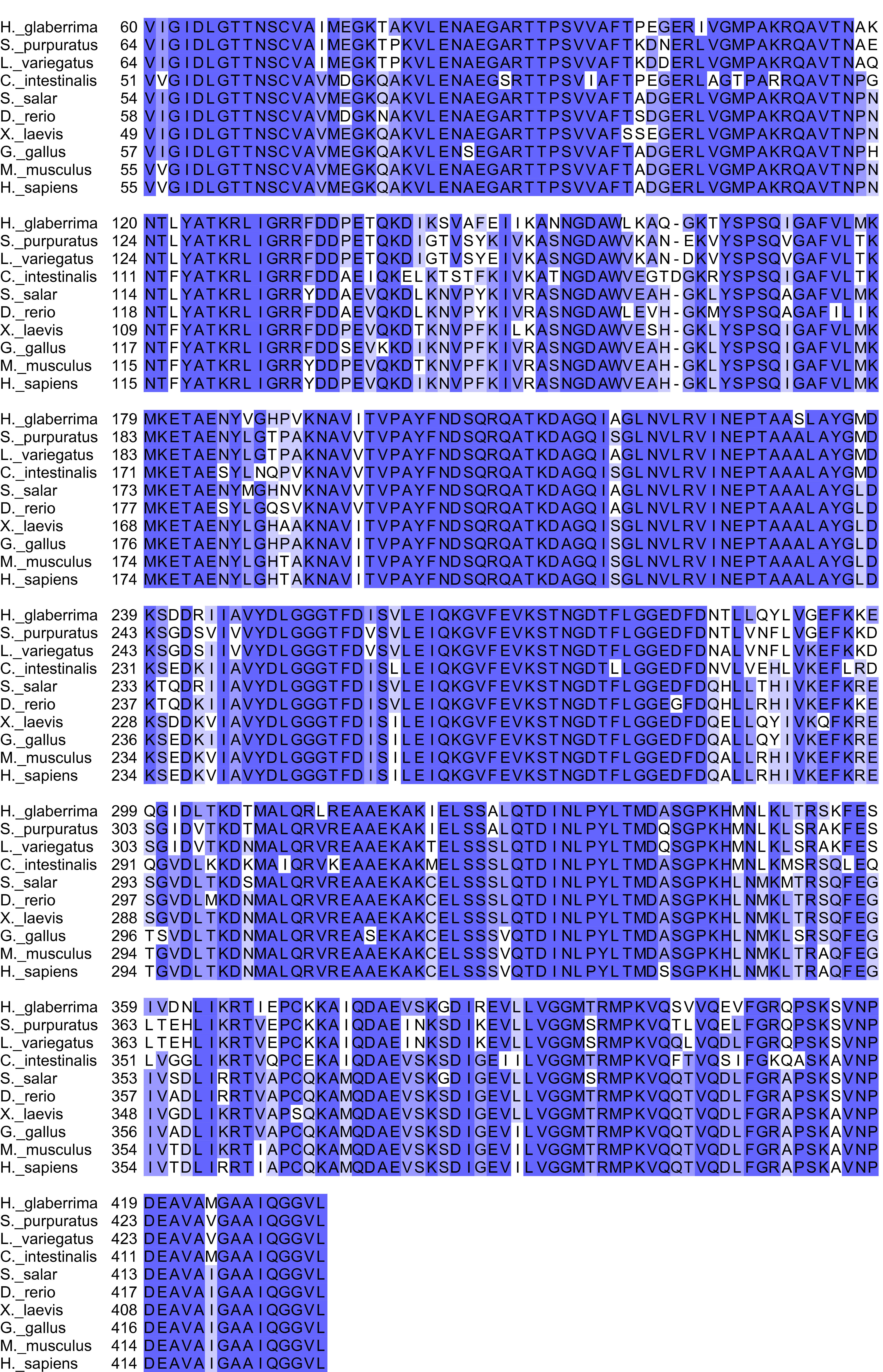

Supplement: Additional file 4 — Alignment of the ATPase domain of mortalin from H. glaberrima and other deuterostome species. Conservative residues are shaded in blue. For the accession numbers of the sequences used in the alignment, see Additional File 6. [file 1471-213X-10-117-S4.TIFF]

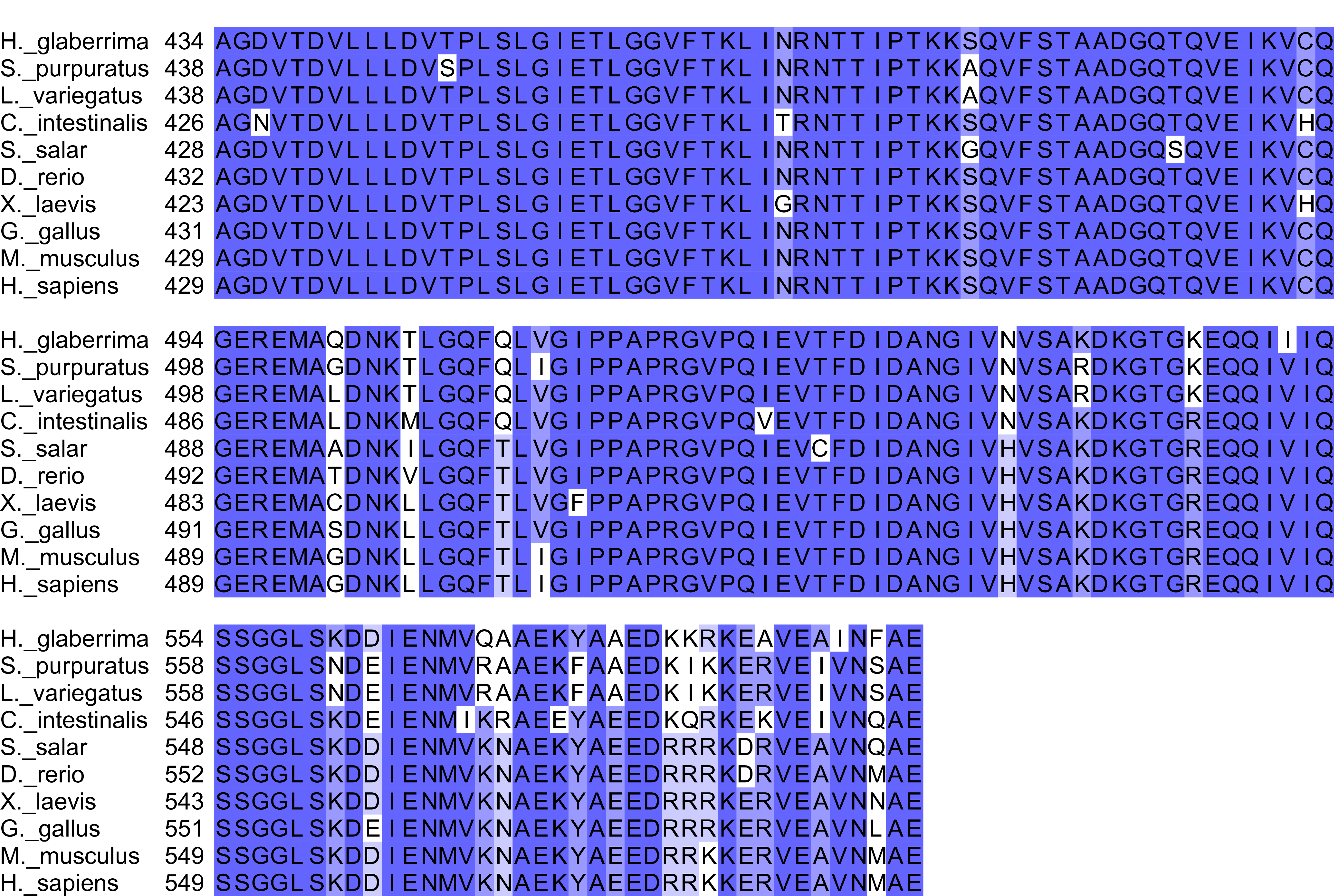

Supplement: Additional file 5 — Alignment of the substrate-binding of mortalin sequences from H. glaberrima and other deuterostome species. Conservative residues are shaded in blue. For the accession numbers of the sequences used in the alignment, see Additional File 6. [file 1471-213X-10-117-S5.TIFF]

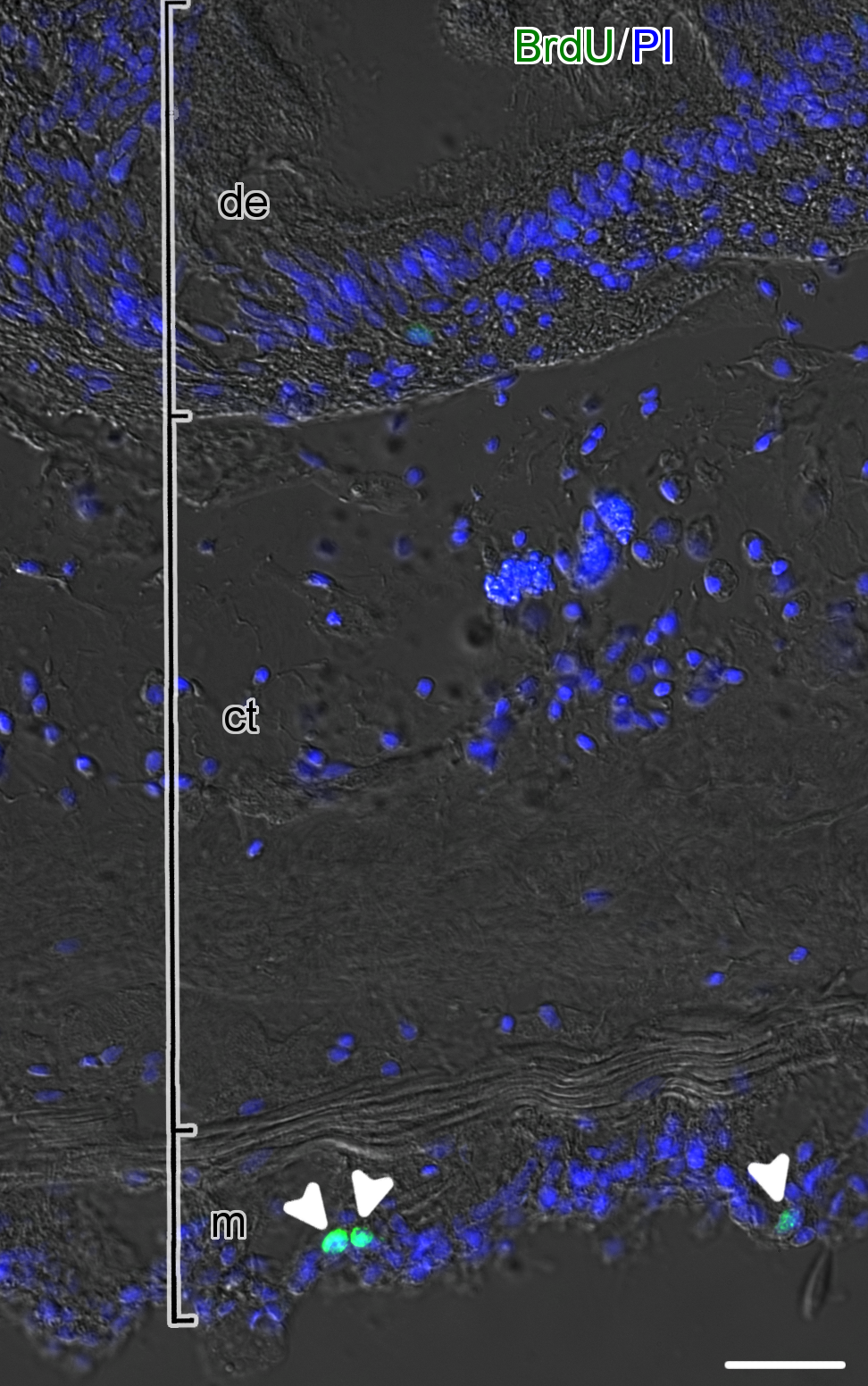

Supplement: Additional file 7 — BrdU-positive cells (green) in the apical region of the mesothelium of the esophagus in a non-eviscerated individual of H. glaberrima. Arrowheads show BrdU-positive cells. Nuclei were stained with propidium iodide and are shown in blue. ct - connective tissue layer; de - digestive (luminal) epithelium; m - mesothelium. Scale bar = 25 μm. [file 1471-213X-10-117-S7.TIFF]
