# Supplementary material for: Visceral regeneration in a sea cucumber involves extensive expression of survivin and mortalin homologs in the mesothelium
Source: BMC Dev Biol. 2010 Nov 29;10:117. doi: 10.1186/1471-213X-10-117 (PMC3013081; doi:10.1186/1471-213X-10-117)
Supplement: Additional file 3 — The overall similarity between the deduced amino acid sequence of H. glaberrima survivin and survivin orthologs of other deuterostomes. [file 1471-213X-10-117-S3.PDF]

Additional File 3.

The overall similarity between the deduced amino acid sequence of *H. glaberrima* survivin and survivin orthologs of other deuterostomes

**Species**	**% identity**	
	**Entire protein**	**BIR Domain**
Strongylocentrotus purpuratus [RefSeq: XP_001175578.1]	53.4	66.7
Saccoglossus kowalevskii [RefSeq: XP_002733289.1]	53.4	69.2
Salmo salar [GenBank: ACI66178.1]	34.7	48.7
Danio rerio [UniProt: Q90WU9]	36.1	48.7
Xenopus laevis [UniProt: Q8JGN5]	37.5	57.7
Gallus gallus [UniProt: Q9DDK0]	41.7	56.4
Mus musculus [UniProt: O70201]	45.1	56.4
Homo sapiens [UniProt: O15392]	43.8	57.7
