# Supplementary material for: Visceral regeneration in a sea cucumber involves extensive expression of survivin and mortalin homologs in the mesothelium
Source: BMC Dev Biol. 2010 Nov 29;10:117. doi: 10.1186/1471-213X-10-117 (PMC3013081; doi:10.1186/1471-213X-10-117)
Supplement: Additional file 6 — The overall similarity between the deduced amino acid sequence of H. glaberrima mortalin and mortalin orthologs of some other deuterostomes. [file 1471-213X-10-117-S6.PDF]

Additional File 6.

The overall similarity between the deduced amino acid sequence of *H. glaberrima* mortalin and mortalin orthologues of other deuterostomes

Species	% identity
	Entire protein
Strongylocentrotus purpuratus [RefSeq: XP_781277.1]	69.2
Lytechinus variegatus [UniProt: A0FKR3]	69.4
Ciona intestinalis [RefSeq: XP_002121036.1]	63.5
Salmo salar [UniProt: C0HAF8]	66.6
Danio rerio [UniProt: Q7ZYY3]	65.9
Xenopus laevis [UniProt: Q7ZX34]	67.2
Gallus gallus [UniProt: Q5ZM98]	66.8
Mus musculus [UniProt: P38647]	65.5
Homo sapiens [UniProt: P38646]	65.6
